# A systems review of grain proteins in rice and barley: biosynthesis, regulation, and impact on end-use quality

**DOI:** 10.3389/fpls.2025.1658144

**Published:** 2025-11-21

**Authors:** Essam ElShamey, Xiaomeng Yang, Jiazhen Yang, Li Xia, Yawen Zeng

**Affiliations:** 1 Biotechnology and Germplasm Resources Institute, Yunnan Academy of Agricultural Sciences, Kunming, China; 2 Rice Research Department, Field Crops Research Institute, Agricultural Research Center, Giza, Egypt

**Keywords:** protein, rice, barley, gene regulation, diet, nutraceuticals, functional foods

## Abstract

Grain proteins in cereal crops play a crucial role in determining both the nutritional value and end-use quality of food products. This systematic review comprehensively examines the biosynthetic pathways, regulatory mechanisms, and functional impacts of storage proteins in rice (*Oryza sativa*) and barley (*Hordeum vulgare*), two of the world’s most important staple crops. Rice and barley are significant sources of bioactive proteins, which are fundamental to their nutritional value and health promoting properties. The major storage proteins, including glutelins, prolamins (with hordeins being the major type in barley), and globulins, are synthesized under the regulation of key transcription factors like *RISBZ* and *RPBF* in rice and *BLZ1/2* in barley. These proteins provide essential amino acids and are a source of bioactive peptides with demonstrated anti-hypertensive, immunomodulatory, and cholesterol-lowering activities. Furthermore, the review considers the influence of genetic and environmental factors on protein profiles. The health implications of rice and barley proteins are discussed, underscoring their potential in functional foods and nutraceuticals. Future perspectives highlight the promise of metabolic engineering and precision breeding for the biofortification and nutritional enhancement of these vital cereals.

## 1 Introduction

Cereal grains are primary sources of dietary protein, particularly in developing countries where animal protein may be less accessible (Borrelli and Ficco, 2025). Rice and barley, despite their differences in cultivation and consumption patterns, contribute significantly to global protein intake. Rice (*Oryza sativa*) was first domesticated from its wild ancestor over 8,000 years ago in the Pearl River valley region of ancient China, with a separate origin for African rice (*Oryza glaberrima*) in the Niger River Delta, establishing it as a foundational crop that would go on to feed a significant portion of the global population. The grain’s exceptional nutritional value is primarily derived from its status as an excellent source of complex carbohydrates, which provide essential energy, but its most crucial nutritional elements include its protein content, which contains a small amount of lysine; valuable B vitamins, especially thiamine (B1); and important minerals like manganese and selenium, though brown rice retains significantly higher amounts of fiber, vitamins, and minerals due to the preservation of its nutrient-rich bran and germ layers (El Sayed et al., 2021), while barley is widely used in animal feed, brewing, and human nutrition in various regions (Timilsina et al., 2025). Proteins in these cereals play vital roles in nutrition, food processing, and industrial applications (Borrelli and Ficco, 2025). However, their protein quality differs due to variations in amino acid composition, digestibility, and anti-nutritional factors. Protein as a functional component in rice and barley refers to the bioactive compounds, nutrients, and other elements that contribute to the health benefits, nutritional value, and functional properties of these cereals.

In this review article, we discuss the key to proteins as one of the most important functional components in both rice and barley and their potential health benefits (Yang et al., 2023; Li et al., 2022; Zeng et al., 2016; Elshamey et al., 2025b, 2025). Proteins are essential macronutrients that play a critical role in human nutrition and plant physiology. In cereal grains such as rice (*Oryza sativa*), proteins are not only a source of dietary amino acid but also contribute to the functional properties of these grains (Poutanen et al., 2022), including their texture, processing quality, and health benefits. This review provides a detailed analysis of the types, structures, and functional roles of proteins in rice and barley, highlighting their nutritional significance, technological application, and potential for improving human health and agricultural sustainability (Zeng et al., 2020, 2017, 2024; Xia et al., 2023).

People with food allergies can use rice proteins because they are hypoallergenic and readily digested. They contain essential amino acids, though lysine is limited (Jayaprakash et al., 2022) (Meza et al., 2024). Rice contain B vitamin (e.g., thiamine and niacin) and minerals like magnesium, phosphorus, and selenium (Hoque et al., 2023). These nutrients support metabolic, bone health, and immune function. Found in rice bran, Phytosterols assist in lowering cholesterol levels and minimizing the risk of cardiovascular disease (Liu et al., 2023).

Barley contains vitamin E compounds, which have antioxidant properties and protect cell membranes from oxidative damage (Dabina-Bicka et al., 2011). Barley proteins are in essential amino acids, particularly lysine, which is often limited in other cereals. They contribute to muscle repair and overall growth (McElroy et al., 1949). Barley is a good source of magnesium, phosphorus, iron, and zinc, which are essential for both health, energy production, and immune function (Rubene and Kuka, 2006). Also, barley contains lignans, which are phytoestrogens with potential anticancer and cardiovascular benefits.

## 2 Protein-related genes in rice (Oryza sativa)

**Figure 1:**
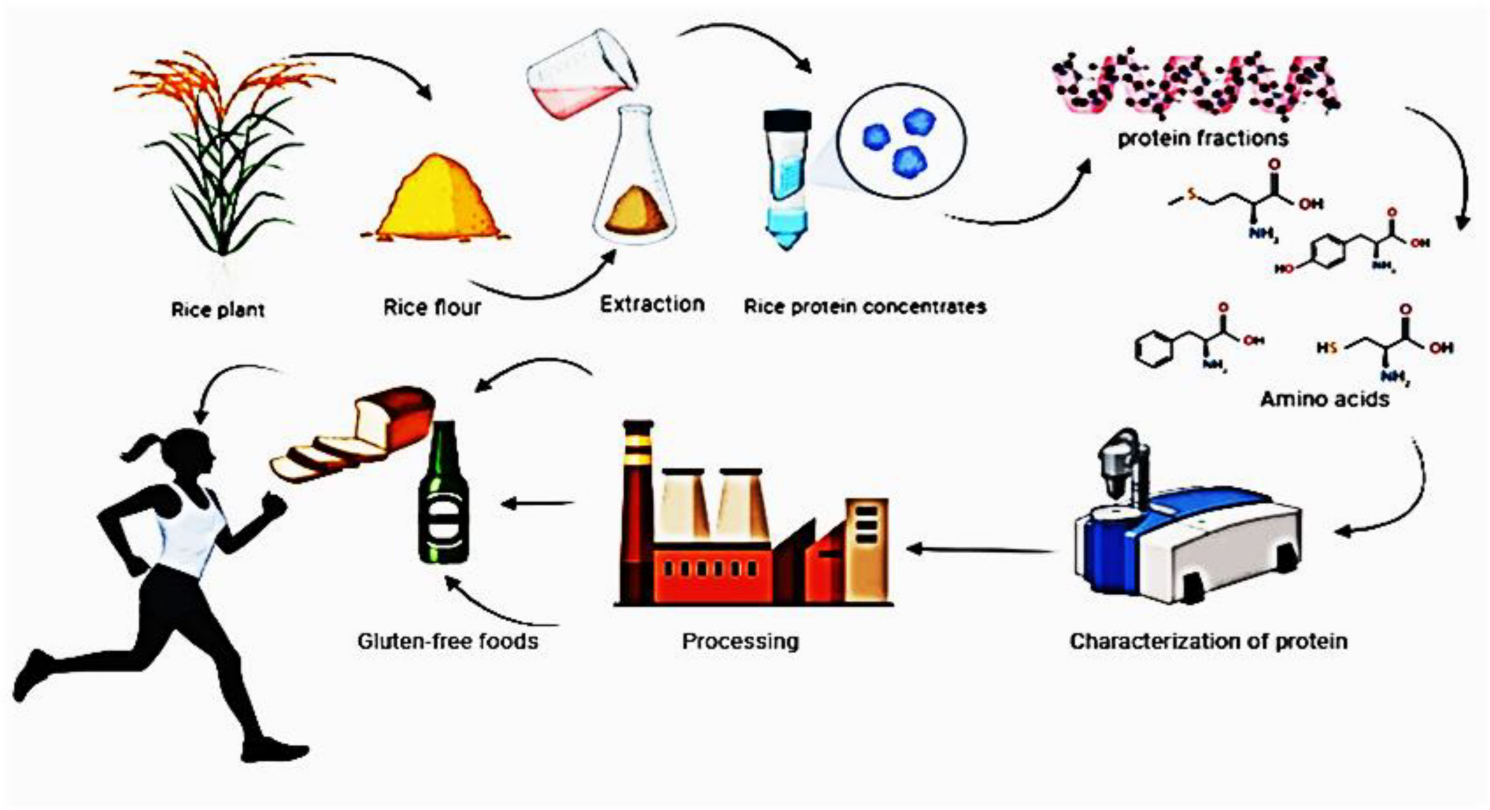
Life cycles of rice protein and its importance to the human body.

Proteins are fundamental components of all living organisms, serving as building blocks for tissues, enzymes, and signaling molecules. In cereal grains, proteins are stored primarily in the endosperm and play a key role in seed development, germination, and stress responses (Müntz et al., 2001). Rice (Indica type or Japonica type), the most widely cultivated cereal crops, are important sources of dietary protein, particularly in regions where animal protein is scarce. This review explores the composition, functionality, and applications of proteins in rice, the life cycles of rice protein and its importance for human body are shown in **Figure 1**, emphasizing their role as functional components in food systems and human health. The interaction and differences in gene loci associated with protein biosynthesis in rice (*Oryza sativa*) involve distinct metabolic pathways, genetic regulation, and physiological functions. Below is a breakdown of their key aspects:

The protein content and quality in rice are determined by a complex interplay of genetic, biosynthetic, and regulatory mechanisms. Rice proteins are primarily classified into four major groups based on solubility: glutelins (the most abundant, 60-80% of total protein), prolamins (alcohol-soluble, 20-30%), globulins (salt-soluble, 5-10%), and albumins (water-soluble, minor fraction). The expression of these proteins is tightly regulated by specific genes, influencing both nutritional value and functional properties.

### 2.1 Storage proteins

Storage protein-related genes in rice play a crucial role in determining grain quality, nutritional value, and digestibility. These genes, such as those encoding glutelins, prolamins, and globulins, regulate protein synthesis and accumulation in rice seeds, influencing both yield and consumer acceptability. Advances in genetic and molecular studies have enabled the identification and manipulation of these genes to improve protein content, amino acid balance, and allergen reduction. Understanding the expression and regulation of storage protein genes provides valuable insights for bio-fortification efforts and the development of high-quality rice varieties to meet global nutritional demands. Glutelins (e.g., *GluA1*, *GluB4*, *and GluC*) are encoded by multigene families, crucial for seed storage proteins (Xinkang et al., 2023; Ram et al., 2021). Prolamins (e.g., *13kDa prolamin*, *RM1*) are minor storage proteins regulated by specific transcription factors (Pham et al., 2024).

### 2.2 Regulatory genes

Regulatory genes play a crucial role in controlling growth, development, and stress responses in rice (*Oryza sativa*). These genes, including transcription factors (TFs), kinases, and epigenetic regulators, orchestrate complex molecular networks that influence traits such as yield, disease resistance, and abiotic stress tolerance. Advances in genomics and CRISPR-based gene editing have enabled precise manipulation of regulatory genes, offering promising avenues for rice improvement. Understanding these genes enhances our ability to develop climate-resilient, high-yielding rice varieties, contributing to global food security. Also, research should focus on uncovering novel regulatory mechanisms and translating findings into sustainable agricultural practices. The genes RISBZ1 and RPBF are basic leucine zipper (bZIP) TFs that regulate storage protein gene expression (Thakur et al., 2021; Cao et al., 2022; Qiu et al., 2023); OsPDIL1–1 are affects protein disulfide isomerase and influencing protein folding in the endoplasmic reticulum (Zhao et al., 2023a; Chen et al., 2024; Zhao et al., 2023b).

### 2.3 Biotechnological & breeding implications

Proteins in rice are a dynamic and intricate process with significant implications for biotechnology and breeding. By leveraging genetic engineering and advanced breeding strategies, it is possible to develop rice varieties with enhanced nutritional benefits and stress resilience. For some biotechnological implications; protein-enriched rice: targeting *Glu* and *Prolamin* genes to improve nutritional quality (Jukanti et al., 2025; Badoni et al., 2024).

Protein-related loci primarily govern seed nutrition and storage. Their interaction lies in shared precursors and regulatory networks, making them important targets for improving rice nutritional and stress-resistant traits. These proteins (glutelins, prolamins, globulins, and albumins) are sequestered within protein bodies (PBs) in the rice endosperm, yet their inherent deficiency in essential amino acids like lysine, threonine, and tryptophan limits rice’s dietary value. The strategy to optimize the genes through biotechnological approaches to enhance proteins as fellow; first, gene-centric enhancement involves the endosperm specific overexpression of lysine-rich protein genes, such as those encoding globulins or modified amylases, using strong promoters like (GluB-1). Crucially, this is coupled with the simultaneous silencing of the major endogenous prolamin genes (e.g., *RP10, RP13*) through RNA interference (RNAi) or CRISPR/Cas9-mediated repression. This dual approach not only increases total protein content but fundamentally rebalances the amino acid profile by suppressing the synthesis of lysine-poor prolamins and creating a metabolic sink for nitrogen and amino acids to be redirected toward the synthesis of nutritionally superior proteins. Second, post-translational and subcellular optimization addresses the inherent bottlenecks in protein accumulation. This entails co-expressing molecular chaperones, such as Binding Protein (BiP), to alleviate endoplasmic reticulum (ER) stress and improve the folding and trafficking of recombinant proteins, thereby preventing their degradation. Furthermore, leveraging protein targeting signals to divert novel storage proteins to specific protein bodies (e.g., type II PBs) can minimize unintended interactions with native storage proteins and optimize packaging within the starchy endosperm. Third, systemic metabolic engineering ensures the substrate availability for enhanced protein synthesis. This includes modulating the endosperm’s free amino acid pool by overexpressing key biosynthetic enzymes in the aspartate pathway, a primary route for lysine and threonine biosynthesis while also downregulating catabolic enzymes like lysine ketoglutarate reductase (LKR/SDH) to prevent lysine degradation. The integration of these approaches represents a paradigm shift from singular gene manipulation to the holistic redesign of the endosperm’s proteome and metabolome. The convergence of advanced genomics, precise genome editing, and synthetic biology promises the development of a new generation of “high-protein rice” that serves as a complete nutritional source, contributing significantly to global food and nutritional security (Sinaga et al., 2025).

### 2.4 Protein composition in rice

**Figure 2:**
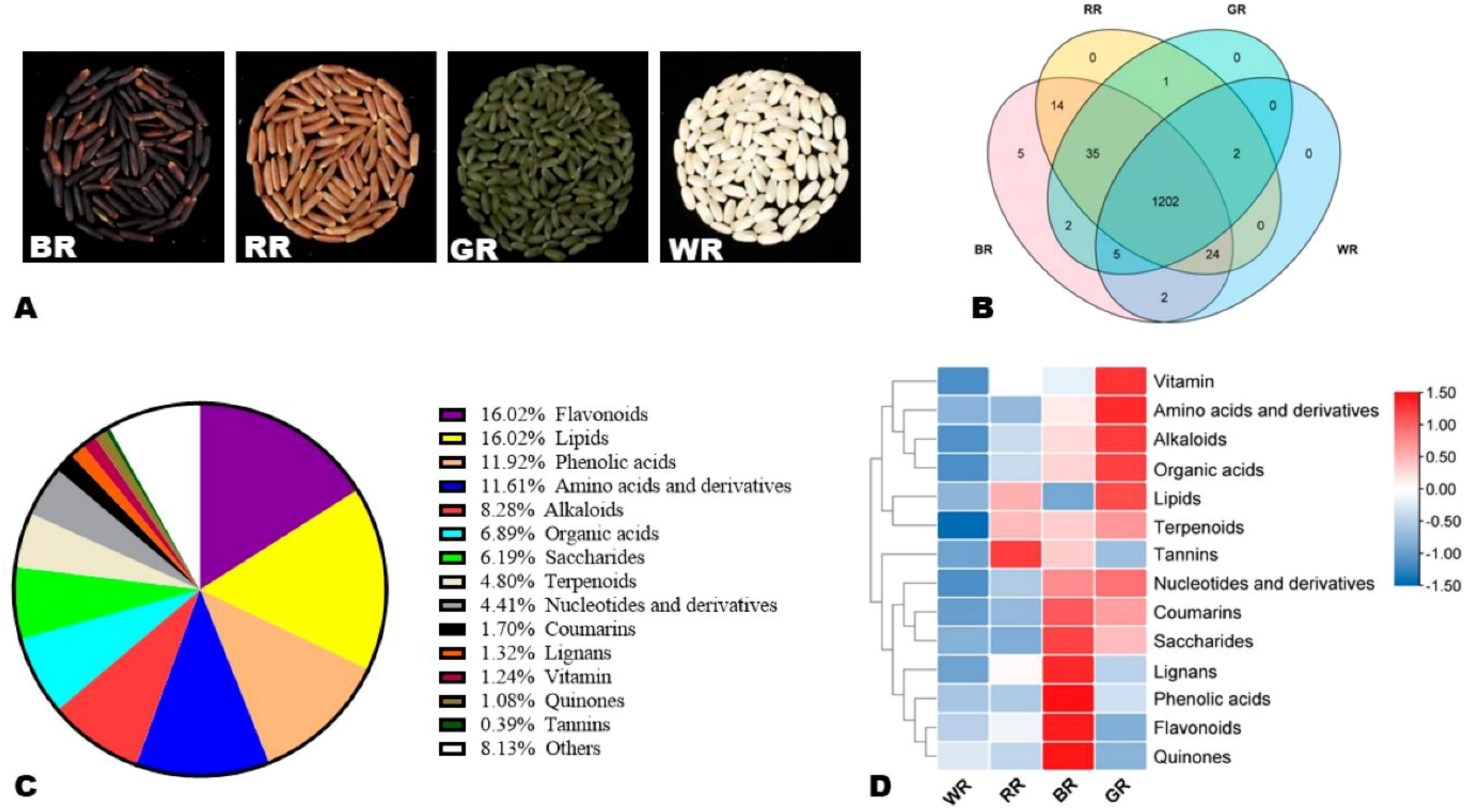
The distinct colored rice grains (brown rice, red rice, green rice, and white rice) morphology and metabolic variations.

The protein composition of rice varies depending on genetic factors, environmental conditions, and post-harvest processing. This discussion explores the key aspects of rice protein, including its content, quality, and nutritional implications. Rice grains typically contain 6–9% protein by weight, which is lower than other cereals like wheat (10–15%) and maize (8–11%). However, the protein content can vary significantly among different rice varieties; brown rice retains the bran layer, which contains higher protein levels compared to polished white rice. Specialty rice varieties, such as black or red rice, may have slightly elevated protein content due to genetic differences, high-protein rice mutants have been developed through breeding programs to enhance nutritional value. The polishing process does not significantly reduce the absolute amount of protein in the rice grain. Instead, it dramatically increases the proportion (percentage by weight) of protein in the final polished white rice. This counter intuitive result occurs because polishing removes other components (primarily fats, fibers, and minerals) at a much faster rate than it removes protein, causing the remaining protein to become more concentrated. The structure of the grain; husk and it is the inedible, protective outer shell, always removed to produce brown rice. Bran layer is the multi-layered, edible outer coating that is removed during polishing, and it is rich in dietary fiber (especially insoluble fiber), lipids (fat and oils), vitamins (B-complex vitamins like thiamine, niacin, B6), minerals (magnesium, phosphorus, potassium, zinc, iron), and bioactive compounds (antioxidants like gamma-oryzanol). Germ (embryo) is the nutrient-dense “heart” of the seed, packed with B vitamins, lipids, and some protein. It is also removed during polishing. Endosperm is the inner, starchy core that makes up most of the white rice grain. Its primary purpose is to provide energy for the germinating plant. It consists mainly of starch (75-80% of white rice by weight), protein (6-7% of white rice by weight), and traces of vitamins and minerals. Crucially, the protein in rice is not evenly distributed. It is higher in the outer layers (the bran and aleurone layer) and decreases towards the center of the endosperm. Polishing, or milling, is a mechanical abrasion process. The brown rice grains are passed through an abrasive roller or friction mill that literally scrapes and scrubs away the bran layer and the germ. Brown rice (100% whole grain with bran and germ intact), while white rice (primarily just the starchy endosperm). Rice bran (the collected dust from the polishing, which is itself a valuable product used for oil extraction and animal feed). The degree of polishing can be controlled. A light polish will remove less bran, resulting in a slightly tan-colored rice, while a heavy polish creates bright white, fully milled rice. The detailed impact on protein, the nutritional transformation using a hypothetical 100g sample of brown rice, contains; starch about 75g and concentrated in the endosperm, protein about 7.5g and higher concentration in the bran layer, lipids about 2.5g and almost exclusively in the bran and germ, and the rest are dietary fiber and minerals and almost exclusively in the bran layer as shown in **Figure 2** (Reddy et al., 2017; Huang et al., 2020).

Polishing removes approximately 8-10% of the brown rice’s weight. This removed material (the bran) is not just one thing; it is disproportionately made up of specific components; about 90% of the lipids (fats) are removed, 80-90% of the dietary fiber is removed, 70-80% of the minerals (ash) are removed, 50-60% of the B vitamins are removed, and only 15-25% of the total protein is removed. Finally, the 100g of brown rice becomes about 92g of white rice. For protein tracking, protein in brown rice is about 7.5g, while protein is lost in bran around 1.3g (assuming 20% of protein is removed), and the remaining protein in white rice is 6.2g. The protein loss is less significant, and the removal of other components is more dramatic. A more accurate representation from agricultural studies shows a typical brown rice protein content is 7.1-7.9% while a typical white rice protein content is 6.3-6.9%. Therefore, while the absolute amount of protein per grain decreases slightly, its relative contribution to the dry weight of the remaining material increases. It becomes more concentrated because the low-protein components (fats, fibers) have been stripped away (Zhao et al., 2025). While concentration might change slightly, the type of protein remains the same. The bran contains a different protein profile, including albumin and globulin, which are considered higher quality proteins. The endosperm protein is primarily glutenin and prolamin (oryzenin), which is a less complete protein. Therefore, while you may have a similar concentration, you lose some of the higher quality protein fractions. Polishing removes a disproportionate amount of the essential amino acid Lysine. Lysine is already the limiting amino acid in rice (the one in shortest supply). Polishing further reduces the overall protein quality of white rice by lowering its Lysine content. The most significant loss is not protein but micronutrients. The bran layer is an incredible source of vitamins and minerals. The loss of thiamine (B1) is so severe that in populations it is reliant on white rice, it caused the disease Beriberi. This is why most white rice is now enriched or fortified synthetic vitamins are added back to the surface of the polished grain to compensate for these losses. Therefore, while brown rice is nutritionally superior overall due to its full spectrum of micronutrients and fiber, white rice ends up with a macronutrient profile that is marginally higher in starch and protein percentage by weight, albeit with a less optimal protein quality (Gul et al., 2015; Moongngarm et al., 2012).

Rice amino acids are classified based on their solubility and functional properties (**Table 1**); glutelins: 60–80% of rice’s total protein composition is made up of the most prevalent storage proteins (Xinkang et al., 2023). They are highly digestible and contribute to the nutritional quality of rice. Prolamins are minor components in rice, making up 5-10% of total protein (Muench et al., 1999), they are less soluble and have limited nutritional value due to low lysin content, albumins and globulins; these water-and salt-soluble proteins are rich in essential amino acids and play a role in metabolic processes during seed development (Aragão et al., 2015).

**Table 1:** The essential and non-essential amino acids in rice by g/100g.

	Amino acids	Rice Protein (g/100g)	References
Essential amino acids	Methionine	0.65-3.49	(Amagliani et al., 2017; Balindong et al., 2018; Wang et al., 2018; Małecki et al., 2021; De Souza et al., 2016; Braspaiboon et al., 2020)
	Histidine	1.19-3.49	
	Threonine	2.09-5.06	
	Cyctine	0.13-3.42	
	Valine	3.78-6.80	
	Leucine	5.30-9.51	
	Isoleucine	2.69-5.18	
	Phenylalanine	3.5-6.30	
	Tyrosine	1.33-6.0	
	Lysine	2.2-6.24	
Non-essential amino acids	Alanine	3.69-6.20	(Singh and Sogi, 2018; Li et al., 2020a; Jayaprakash et al., 2022; Cañizares et al., 2024; Leal et al., 2021)
	Serine	2.96-5.64	
	Glutamic acid	13.36-22.42	
	Proline	2.70-14.88	
	Aspartic acid	8.10-10.98	
	Glycine	4.21-5.98	
	Arginine	5.30-984	

**Figure 3:**
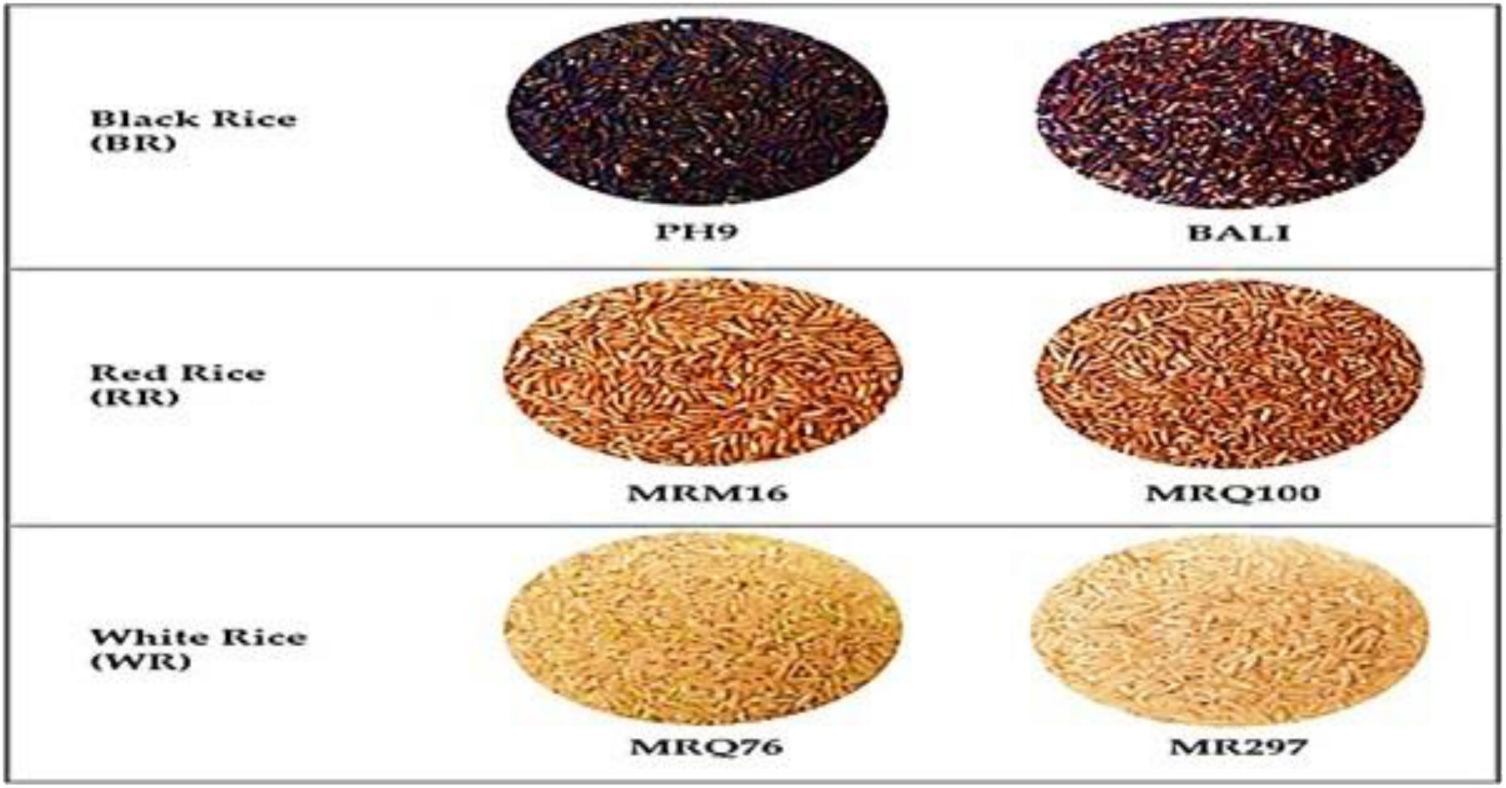
A selection of pigmented and non-pigmented rice cultivars’ dehusked grains.

Total protein content, rice typically contains 7-9% protein by weight, depending on the variety and growing conditions, pigmented rice varieties are shown in **Figure 3** (Fageria, 2007; Sew et al., 2023); white rice: around 7% protein (polished grains have lower protein content due to the removal of the bran and germ), brown rice; around 8-9% protein (higher due to the retention of the bran layer), and protein distribution; most rice proteins are found in the endosperm, with smaller amounts in the bran and germ.

While rice is not a complete protein source on its own, its widespread consumption makes it a vital contributor to global protein intake. Understanding and improving rice protein composition can enhance its nutritional value, particularly in populations reliant on rice-based diets. Future research should focus on biofortification, protein enrichment, and optimizing processing methods to maximize the benefits of rice protein in human nutrition.

## 3 Protein-related genes in barley

In addition to being the most functional meal for the human body’s cellular nutrition and detoxification, barley grass also has the highest levels of biologically active compounds for a variety of positive health impacts. Vitamins and the tryptophan mechanism in barley grass enable it to combat over 20 chronic disorders. Important food assistance, Barley grass meal can satisfy the WHO’s low sodium (<2g) and high potassium (>3.5g) daily targets (Zeng et al., 2020; Yang et al., 2016). Barley grain contains 15 functional components that can prevent 11 chronic diseases, while barley grass contains over 30 functional components that can prevent over 20 chronic diseases. The LTP2 gene, which mediates intracellular lipid transport and is involved in abiotic stress responses, increased the production of sterols in barley (Kempa et al., 2024). All living things are made up of proteins, which are essential building blocks for tissues, enzymes, and signaling molecules. Proteins in cereal grains are mostly found in the endosperm and are essential for stress reactions, germination, and seed development. Especially in areas where animal protein is limited, barley, a popular and extensively grown cereal crop, provides a significant source of dietary protein. With a focus on their significance as useful elements in food systems and human health, this review examines the makeup, functions, and uses of barley proteins. Barley (*Hordeum vulgare* L.) is a crucial cereal crop with significant nutritional value, serving as both a food source and a key ingredient in brewing. Two important classes of barley compound storage proteins and flavonoids—play distinct but interconnected roles in grain quality, plant defense, and human health (Ramakrishna et al., 2019; Loskutov and Khlestkina, 2021; Shahidi et al., 2022). Understanding the genetic loci controlling these metabolic pathways and their interactions provides insights for barley improvement.

### 3.1 Storage protein genes

Storage protein genes play a crucial role in plant growth and development by encoding proteins that serve as nutrient reserves, particularly in seeds. These genes are highly regulated and influence the nutritional quality of crops, making them essential targets for agricultural biotechnology. Advances in genetic engineering and molecular breeding have enabled the modification of storage protein genes to enhance crop yield, improve amino acid composition, and reduce allergenic properties. Understanding the expression, regulation, and evolution of these genes provides valuable insights for developing biofortified crops to address global food security challenges. The storage genes included: Hordoindoline (Hina, Hinb) - located on chromosome 5H, these genes affect grain hardness and milling quality (Yanaka et al., 2011); B-hordein (Hor2) - found on chromosome 1H, encoding major storage proteins (Shewry et al., 1983); C-hordein (Hor1) - also on 1H, contributing to prolamin content (Uddin et al., 2014); D-hordein (Hor3) - a minor storage protein locus on 5H (Shewry et al., 1983); and Glutelin genes **-** distributed across multiple chromosomes, influencing dough elasticity (Li et al., 2020b).

### 3.2 Regulatory genes

Regulatory genes in barley play a pivotal role in controlling growth, development, and stress responses by modulating the expression of downstream target genes. These genes, including transcription factors, microRNAs, and epigenetic regulators, influence key agronomic traits such as flowering time, drought tolerance, nutrient uptake, and disease resistance. Advances in genomics and molecular biology have enhanced our understanding of barley’s regulatory networks, providing valuable insights for crop improvement. By leveraging this knowledge, researchers can develop barley varieties with enhanced yield, resilience, and adaptability through targeted genetic engineering or marker-assisted breeding, regulatory genes in barley included; SPA (storage protein activator) - regulates prolamin expression (Albani et al., 1997; Plessis et al., 2023), and BLZ1 (bZIP transcription factor). Binds to the GCN4-like motif in storage protein promoters (Xi and Zheng, 2011; Carbonero et al., 2000).

### 3.3 Protein biosynthesis pathways

In barley (*Hordeum vulgare*), protein regulation biosynthesis is crucial for both agricultural performance and nutritional quality. This review explores the molecular interactions between key proteins (such as chalcone synthase and transcription factors (MYB and bHLH). Advances in omics technologies have elucidated post-translational modifications, protein-protein interactions. Additionally, we discuss genetic and environmental factors influencing these pathways, alongside biotechnological strategies to enhance protein content for improved barley resilience and human health benefits. Understanding these interactions provides a foundation for targeted breeding and metabolic engineering in cereal crops.

proteins particularly enzymes and regulatory factors govern the biosynthesis, modification, and degradation of these metabolites. The metabolic cross-talk between these pathways is mediated by shared intermediates, transcriptional regulators, and post-translational modifications, ensuring coordinated responses to environmental and developmental cues (Yoo et al., 2013; Qian et al., 2019).

#### 3.3.1 The sophisticated enzymatic orchestration of protein biosynthesis in barley

The biosynthesis of proteins in barley (*Hordeum vulgare* L.) is not a simple, linear pathway but a masterfully orchestrated symphony of enzymatic regulation, fine-tuned by genetic, metabolic, and environmental cues. This complex process, fundamental to the grain’s nutritional and functional quality especially for malting and brewing, is governed at multiple tiers, from transcriptional initiation to co-translational folding and post-translational modification. A deep examination reveals that enzymatic control is the central mechanism integrating these tiers into a coherent and responsive system. At the most fundamental level, regulation begins with the enzymes of the transcriptional machinery. Transcription factors (TFs), which are themselves proteins whose activity is often regulated by phosphorylation (catalyzed by kinases and phosphatases), bind to specific promoter elements of storage protein genes (e.g., hordeins) (Escalante et al., 2012; Islam et al., 2021). The expression of these TFs is the first enzymatic gate, determining the potential for protein synthesis. Hormonal signals, particularly gibberellic acid (GA) and abscisic acid (ABA) during seed development, activate cascades of enzymatic reactions that ultimately modulate these TFs, creating a temporal switch from nitrogen mobilization to storage protein accumulation.

**Figure 4:**
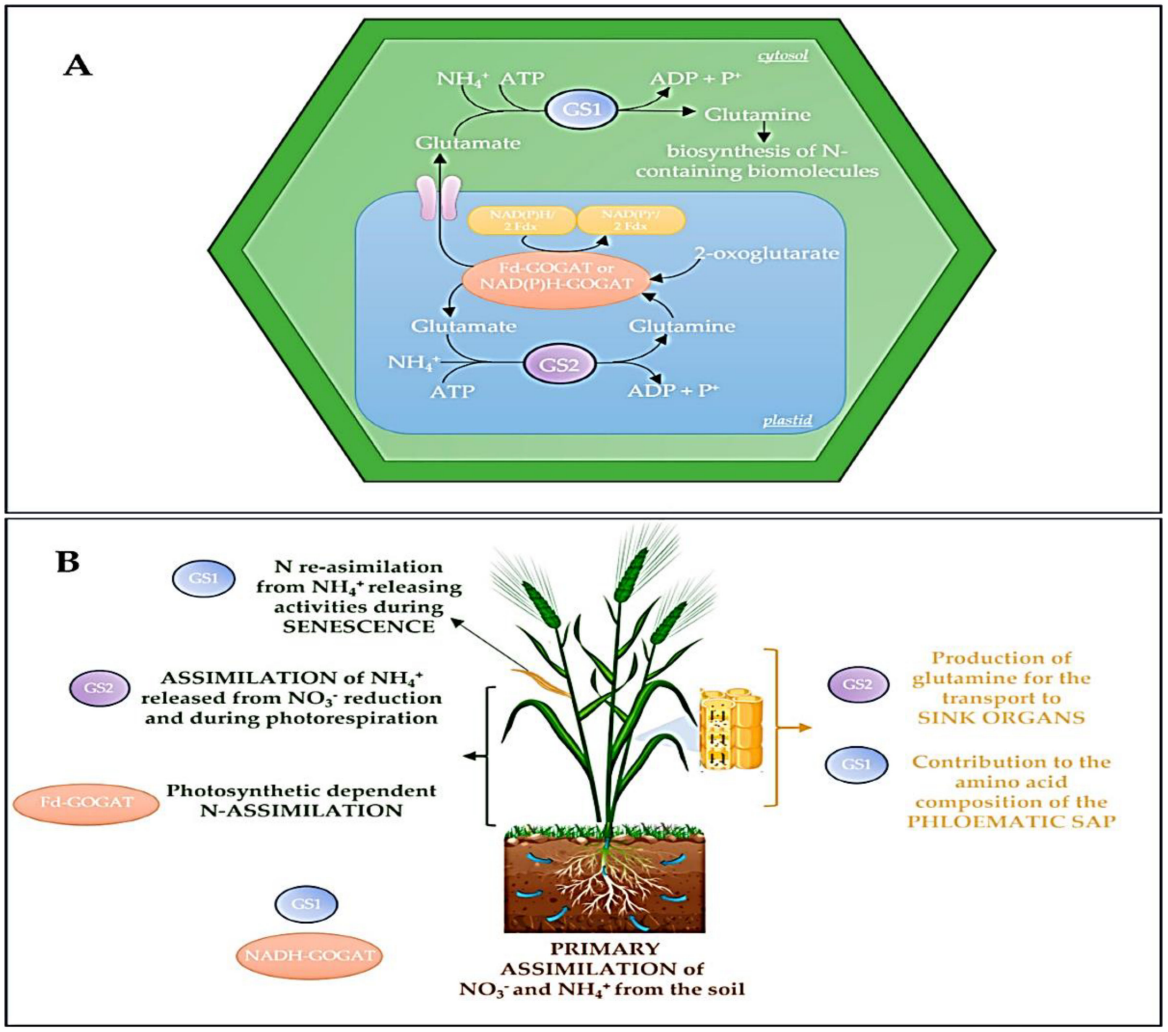
Maps the physical location of the GS-GOGAT cycle in plant cells and describes how the function of its enzymes is determined by their localization. **(A)** a diagram of where this metabolic cycle takes place inside a typical plant cell. **(B)** explains that the different versions (isoenzymes) of the GS and GOGAT enzymes have specialized roles based on their specific locations within the cell.

The commitment of resources is then enzymatically controlled through amino acid biosynthesis. Enzymes like glutamine synthetase (GS) and glutamate synthases (GOGAT) are pivotal in assimilating inorganic nitrogen into organic amino acids, the very building blocks for protein synthesis as shown in **Figure 4**. The activity of these enzymes directly determines the pool of available substrates. This metabolic regulation ensures that protein biosynthesis is tightly coupled to the plant’s nitrogen status and photosynthetic capacity, preventing futile cycles and energy waste. The core process of translation is itself an enzymatic marvel. The ribosome, a ribozyme with intrinsic catalytic activity, is the central workbench. However, its function is heavily dependent on auxiliary enzymes. Aminoacyl-tRNA synthetases perform the critical first step of translation fidelity, ensuring each tRNA is charged with its correct amino acid through an energy-dependent enzymatic reaction. Furthermore, a suite of translation factors (e.g., eIF4E, eIF2, EF-Tu, EF-G), whose activities are regulated by GTP hydrolysis, govern the initiation, elongation, and termination phases. The phosphorylation of these factors, particularly by target of rapamycin (TOR) kinase and SNF1-related kinase (SnRK1), acts as a master throttle, upregulating translation under energy-sufficient conditions and downregulating it during stress. Perhaps the most crucial layer of regulation for defining final protein functionality occurs post-translationally. Molecular chaperones, such as heat shock proteins (HSP70, HSP90) and chaperonins, are ATP-hydrolyzing enzymes that facilitate the correct folding of nascent polypeptide chains, preventing aggregation and ensuring functional conformation. Simultaneously, the enzymes of the secretory pathway signal peptidases, protein disulfide isomerase (PDI) in the endoplasmic reticulum, and various Golgi-resident glycosyltransferases and proteases modify and sort proteins for their destination. For storage proteins like hordeins, this determines whether they are properly deposited into protein bodies, which is essential for the grain’s storage efficiency and its subsequent behavior during malting.

Finally, the entire system is subject to proteolytic control. The balance between synthesis and degradation is maintained by proteolytic enzymes like caspases during development and a vast array of proteases (e.g., endo-proteases, carboxypeptidases) during germination. The activation of these hydrolytic enzymes during malting is the ultimate expression of enzymatic regulation; the stored proteins, synthesized under one set of enzymatic rules, are systematically broken down by another to release amino acids and peptides for the growing yeast (Caretto et al., 2015; Deng et al., 2019). In summary, the enzymatic regulation of protein biosynthesis in barley is a multi-dimensional, hierarchical control network. It seamlessly integrates genetic instruction (via TF enzymes), metabolic status (via biosynthetic enzymes), translational efficiency (via kinases and translation factors), and post-translational fate (via chaperones and modifying enzymes). This intricate web ensures that protein synthesis is not only efficient but also exquisitely responsive to the plant’s developmental program and external environment. Understanding this regulation is paramount; it provides the foundational knowledge to manipulate barley quality through breeding and biotechnology, aiming for ideal protein profiles that enhance malting performance, boost nutritional value, and ensure resilience in a changing climate. The barley grain, therefore, stands as a testament to the profound role of enzymatic precision in shaping the very fabric of agricultural productivity and end-use quality.

#### 3.3.2 Stress responses and metabolic adaptation

Barley (*Hordeum vulgare* L.), a cereal crop of global importance, is frequently exposed to a multitude of abiotic and biotic stresses that significantly constrain its yield and quality. This review synthesizes current knowledge on the intricate physiological, molecular, and metabolic adaptation mechanisms barley employs to mitigate stress induced damage. Under abiotic duress, such as drought, salinity, extreme temperatures, and nutrient deprivation, barley initiates a complex reprogramming of its metabolic network. This includes the accumulation of key compatible solutes like proline, glycine betaine, and sugars that act as osmolytes and Osmo protectants, the activation of the antioxidant defense system to scavenge reactive oxygen species (ROS), and the production of protective proteins like dehydrins and heat shock proteins (HSPs). Concurrently, hormonal signaling pathways, particularly those involving abscisic acid (ABA), jasmonic acid (JA), and salicylic acid (SA), are pivotal in orchestrating these defense responses. Furthermore, biotic challenges from pathogens and pests trigger distinct metabolic shifts, often involving the synthesis of specialized antimicrobial compounds, phytoalexins, and the reinforcement of physical barriers through lignin and callose deposition. The remarkable genetic diversity within barley germplasm, particularly between wild and cultivated accessions, provides a critical resource for elucidating the genetic basis of these adaptive traits. Understanding the interplay between stress perception, signal transduction, and subsequent metabolic adaptation is paramount for developing novel strategies and breeding more resilient barley cultivars, which is increasingly crucial for ensuring food security in the face of climate change.

### 3.4 Implications for barley breeding

**Figure 5:**
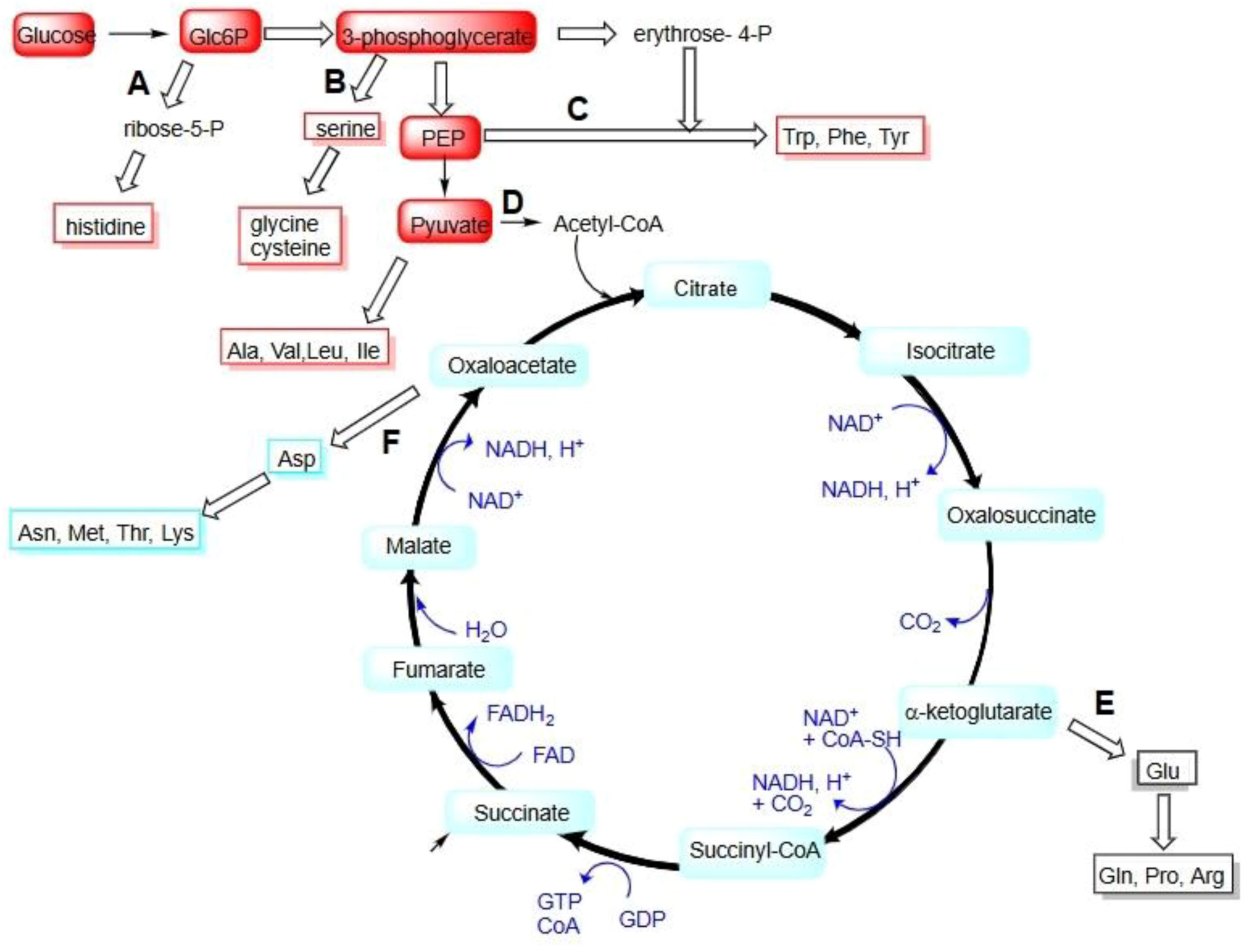
Amino acids biosynthesis pathways.

The gene loci controlling barley protein biosynthesis show both independent regulation and important interactions. While located on different chromosomes and following distinct biosynthetic logic, these pathways compete for precursors and may share some regulatory mechanisms as shown in **Figure 5**. Understanding these relationships enables targeted breeding for improved barley quality traits. Nutritional quality, balancing high protein content with beneficial flavonoids (Solnier et al., 2023). Malting quality, some flavonoids contribute to beer flavor/color while proteins affect foam stability (Deng et al., 2019). Barley (*Hordeum vulgare* L.) is a cornerstone of global agriculture, primarily serving as animal feed and a key raw material for the malting and brewing industries. The nutritional and functional properties of barley grain are predominantly determined by its seed storage proteins (hordeins), which, however, are deficient in essential amino acids like lysine, threonine, and methionine, limiting their nutritional value. Furthermore, the specific composition and quantity of these proteins are critical determinants of malt quality, affecting fermentability, foam stability, and haze formation in beer. This review explores the advanced biotechnological approaches employed to optimize the barley genome to enhance protein content and quality. We focus on strategies aimed at two primary objectives; first is improving nutritional profile for feed and food purposes by increasing essential amino acid content, and second is modulating protein composition to optimize technological properties for malting. Key methodologies discussed include CRISPR-Cas9-mediated gene editing for the precise knockout of specific hordein genes (e.g., C-hordeins) that contribute to amino acid imbalance, and the targeted upregulation of genes encoding lysine-rich proteins. Additionally, we examine transgenic approaches for the introduction and expression of foreign genes encoding high-value proteins or entire biosynthetic pathways for essential amino acids. The integration of functional genomics and high-throughput phenotyping is also highlighted as essential for identifying novel candidate genes and regulatory elements controlling protein synthesis and deposition. By leveraging these sophisticated biotechnological tools, it is possible to redesign barley’s proteome, creating elite cultivars with tailored protein traits that meet the escalating demands for high-quality nutrition and optimized industrial processing (Goedeke et al., 2007; Safhi, 2025).

### 3.5 Protein composition in barley

The most often utilized grain in the brewing sector for making beverages like beer is barley. This review will describe the range of proteins found in barley and examine how they are extracted and used, especially those from brewers’ leftover grain (Jaeger et al., 2021).

#### 3.5.1 Types of proteins

Barley grains typically contain 8–15% protein (dry weight basis), depending on genetic factors, environmental conditions, and agronomic practices. The protein fraction consists of four major groups based on solubility (Osborne classification): hordeins are the major storage proteins in barley, accounting for 35-50% of total protein. They are alcohol-soluble and contribute to the viscoelastic properties of barley dough (Shewry and Darlington, 2024), glutelins like rice, glutelins in barley are important for seed storage and nutritional quality, and both of albumins and globulins are essential amino acids and abundant in these proteins and play a role in stress responses and germination, for barley amino acids as shown in **Table 2**.

**Table 2:** Amino acid contents and composition of barley, barley malt.

Amino acids	Barley	Malt	References
Aspartic acid	0.19	0.17	(Waters et al., 2012; Biel and Jacyno, 2013; Knežević et al., 2007)
Alanine	0.22	0.23	
Γ-aminobutyric acid	2.56	0.01	
Asparagine	0.23	0.33	
Valine	2.56	0.24	
Isoleucine	0.17	0.17	
Tyrosine	0.14	0.14	
Glutamic acid	0.85	0.75	
Lysine	2.52	3.69	
Arginine	0.21	0.23	
Serine	0.12	0.07	
Phenylalanine	0.2	0.21	
Histidine	1.59	1.9	
Leucine	0.3	0.29	
Glycine	0.08	0.06	
Threonine	0.01	0.02	
Total protein (%w/w)	9.65	8.52	

#### 3.5.2 Total protein content

The total protein content in barley is a crucial quality parameter that influences its use in food, feed, and brewing industries. The protein concentration in barley grains typically ranges between 8% and 15%, depending on genetic factors, environmental conditions, agronomic practices, and post-harvest processing. There are many key factors influencing protein content; genetic variability, different barley cultivars exhibit varying protein levels due to inherent genetic traits. High-protein varieties are preferred for animal feed, while low-protein barley is ideal for malting and brewing. Environmental Conditions: soil fertility, nitrogen availability, temperature, and water stress significantly impact protein accumulation. Nitrogen fertilization is a major determinant, as it directly enhances amino acid synthesis and protein formation. Agronomic practices: crop rotation, irrigation, planting density, and harvest timing influence protein content. Late-season nitrogen application tends to increase grain protein but may reduce starch content. Post-harvest processing: storage conditions, drying temperature, and malting processes can alter protein solubility and digestibility.

**Figure 6:**
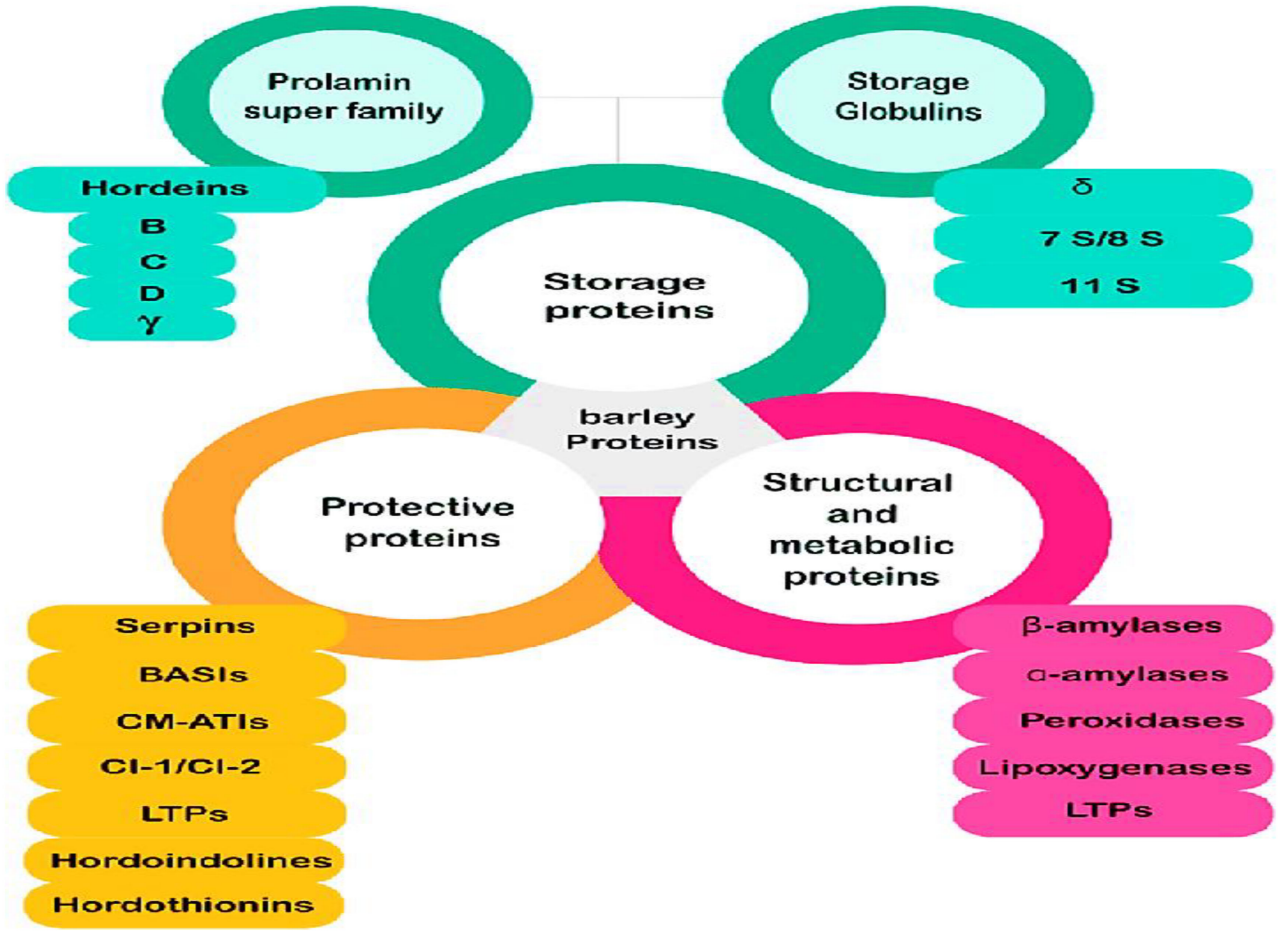
Barley protein classification.

In conclusion, the total protein content in barley is a dynamic trait shaped by both biological and environmental factors. Understanding its regulation is critical for enhancing barley’s role in global food security, animal nutrition, and industrial applications. Barley generally has a higher protein content than rice, ranging from 10-15% protein by weight; hulled barley (10-12%),pearled barley (8-10%) lower due to the outer hull and bran, and for protein distribution barley proteins are concentrated in the endosperm, with significant amounts also present in the bran and germ, barley proteins composition were shown in **Figure 6** (Jadhav et al., 1998; Bahmani et al., 2021).

## 4 Biosynthesis and regulation of proteins in rice and barley

The biosynthesis and regulation of proteins in rice (*Oryza sativa*) and barley (*Hordeum vulgare*) are fundamental processes that determine their growth, development, and adaptation to environmental stresses. Both cereals are vital for global food security, and understanding their protein dynamics provides insights into improving nutritional quality, yield, and stress resilience. The synthesis involves two major stages: transcription (DNA to mRNA in the nucleus) and translation (mRNA to protein on ribosomes in the cytoplasm). The unique aspects in these plants lie in the regulation of these processes, which is orchestrated by a complex interplay of genetic and environmental factors.

### 4.1 Biosynthesis pathway

Protein synthesis in rice and barley follows the central dogma of molecular biology, involving transcription, translation, and post-translational modifications (PTMs). These crops possess complex regulatory networks that modulate gene expression in response to developmental cues and external stimuli. Key enzymes, ribosomal machinery, and chaperones ensure proper protein folding and functionality. Storage proteins, such as glutelins and prolamins in rice and hordeins in barley, are crucial for seed nutrition and have been extensively studied for their role in grain quality. Storage proteins were synthesized during seed development and stored in protein bodies within the endosperm, and metabolic proteins were produced during germination and stress responses to support growth and survival.

### 4.2 Regulation by environmental factors

**Figure 7:**
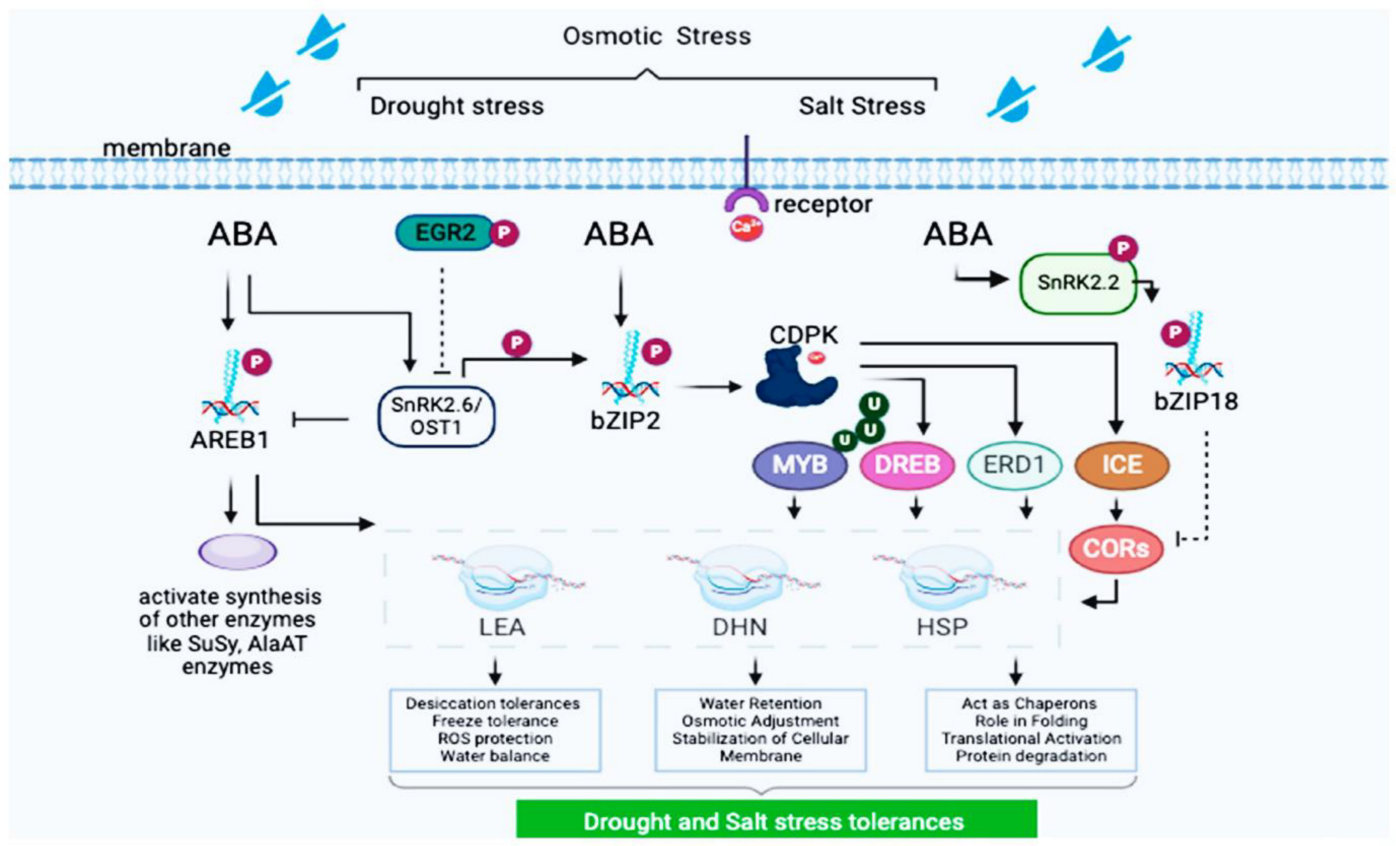
AREB1 and bZIP2-mediated gene networks in ABA-dependent drought and salt stress adaptation. The key differences between rice and barley were summarized as shown in **Table 3**.

The regulation of protein biosynthesis occurs at multiple levels; transcriptional control (TFs) like *bZIP*, *MYB*, and *NAC* families regulate stress responsive and storage protein genes; post-transcriptional modifications are alternative splicing, miRNA-mediated silencing, and RNA stability influence protein abundance; translational and post-translational regulation like Phosphorylation, ubiquitination, and glycosylation fine-tune protein activity and turnover; environmental and hormonal influence are abiotic stresses (drought, salinity) and phytohormones (ABA, gibberellins) modulate protein synthesis pathways as shown in **Figure 7**. While both cereals share conserved protein synthesis mechanisms, differences exist in their storage protein composition and stress adaptation strategies. Barley’s hordeins are major allergens, whereas rice proteins are generally hypoallergenic, making rice a safer dietary staple. Additionally, barley exhibits greater cold tolerance due to unique dehydrins and late embryogenesis abundant (LEA) proteins, while rice has evolved specialized heat-shock proteins (HSPs) for high-temperature resilience. For environmental factors like nitrogen availability protein content in rice and barley is influenced by nitrogen fertilization, with higher nitrogen levels leading to increased protein synthesis, and abiotic stress drought, salinity, and temperature stress can alter protein composition and functionality.

**Table 3:** Key differences and specifics: rice vs. barley.

Feature	Rice	Barley
Major SSPs	Glutelins (acid-soluble, higher lysine), Prolamins (Oryzins, alcohol-soluble, low lysine)	Prolamins (Hordeins - constitute 35-50% of total grain protein, very low lysine)
Protein Bodies	Form two types: PB-I (from ER, containing prolamins) and PB-II (from PSV, containing glutelins).	Primarily one type, derived from the ER and vacuoles, containing hordeins.
Nutritional Goal	Increasing lysine content (as lysine is limiting in prolamin-rich diets). Mutants like opaque mutants have shifted balance from prolamin to higher lysine proteins.	Managing hordein content for malting and brewing. Low-protein barley is desired for brewing, while high-protein barley is for animal feed.
Regulatory Focus	Understanding the balance between glutelin and prolamin synthesis to improve nutritional quality without compromising yield.	Understanding the environmental (esp. heat) regulation of hordein synthesis to ensure consistent quality for malting.

In conclusion, while both rice and barley utilize different storage proteins for seed storage, their nutritional and regulatory goals are shaped by their primary end uses. Rice’s protein composition is split between lysine-poor prolamins and lysine-rich glutelins, leading to a research focus on increasing overall lysine content to improve its nutritional quality for human consumption. In contrast, barley’s protein profile is dominated by lysine-deficient hordeins (prolamins), and its research is not focused on nutrition but rather on managing protein levels for specific industrial applications: low protein for optimal brewing and high protein for animal feed. Consequently, regulatory research for rice aims to balance protein synthesis to improve diet, while barley research seeks to understand environmental impacts on hordein levels to ensure consistent quality for the malting industry.

#### 4.2.1 Comparison of the protein in rice and barley, covering their similarities and differences

While both rice and barley are important cereal grains and sources of plant-based protein, they differ significantly in their protein quality, quantity, and nutritional context. Barley generally offers a superior protein profile due to its higher protein content, better amino acid balance, and the presence of specific beneficial proteins like hordenines. However, the choice between them depends heavily on dietary needs, culinary use, and the rest of your diet (Nitrayová et al., 2018; Zhao et al., 2020).

#### 4.2.2 Similarities between rice and barley protein

- Incomplete protein source, both rice and barley are considered “incomplete” proteins because they lack sufficient amounts of one or more of the nine essential amino acids that the human body cannot produce on its own.
- Limiting amino acid, lysine is the primary limiting amino acid in both grains is lysine. This means lysine is present in the smallest amount relative to human requirements, limiting the body’s ability to use the other amino acids for protein synthesis.
- Complementary protein potential, both can be combined with other plant-based foods to form a “complete” protein. For example, eating rice or barley with legumes (beans, lentils), nuts, or dairy products provides the missing lysine and creates a full amino acid profile.
- Gluten-free status (specific types), while barley contains gluten, brown and white rice are naturally gluten-free. It’s important to note that only specific types of barley (like hulled or pearled) are discussed here; wheat-barley hybrids like triticale are different.

#### 4.2.3 Differences between rice and barley protein

Rice (Oryza sativa) and barley (Hordeum vulgare) are fundamental cereal grains with distinct nutritional profiles, particularly regarding their protein content and quality. The primary differences between them can be categorized into three areas: quantity, quality, and digestibility.

- Firstly, barley generally contains a higher quantity of protein than white rice, with typical values ranging from 10-12% compared to rice’s 6-7%. More significantly, the quality of the protein differs. The nutritional value of a protein is determined by its amino acid profile, specifically its content of essential amino acids. Both grains are limiting in lysine, which is typical for cereals. However, barley often has a more balanced overall amino acid profile and a higher content of certain essential amino acids like threonine compared to rice.
- Furthermore, the digestibility of barley’s protein is complicated by its high fiber content, particularly beta-glucan, which can interfere with protein absorption. In contrast, the protein in white rice is highly digestible due to its minimal fiber content after milling. Consequently, while barley may offer more protein per gram, its net utilizable protein may be lower than its total content suggests. In summary, barley provides a greater total amount of protein, but rice offers protein with superior digestibility. For a complete protein intake, both should be complemented with other protein sources, such as legumes, to compensate for their lysine deficiency.

In conclusion, barley is better if the goal is a higher total protein intake per serving, along with the benefits of immense fiber content for satiety and gut health. It is the more nutritionally dense option overall. Rice is better if they need a highly digestible, low-irritation source of energy and protein (e.g., post-illness), or if you require a gluten-free grain. Its protein, while lower, is efficiently absorbed. For the average healthy individual seeking to maximize protein and overall nutrition from whole grains, barley has a clear advantage over rice in terms of protein content and complementary health benefits. However, both are valuable components of a balanced diet and should be chosen based on individual dietary needs, taste preferences, and meal plans. Combining either with legumes is the key to unlocking their full protein potential.

## 5 Health benefits of protein as functional components in rice and barley

### 5.1 Weight management

Proteins play significant roles as functional components in weight management, offering complementary mechanisms to support healthy body weight. Proteins contribute by enhancing satiety, increasing energy expenditure through thermogenesis, and preserving lean muscle mass during weight loss (Joye, 2019). These bioactive compounds provide a synergistic approach to weight management by promoting metabolic efficiency, reducing fat accumulation, and supporting overall metabolic health. Incorporating protein-rich foods and flavonoid-containing plant sources into a balanced diet may thus be an effective strategy for sustainable weight control and long-term well-being. Rice and barley’s high fiber content encourages fullness, which lowers total caloric intake and helps with weight control (Haque et al., 2025).

### 5.2 Anti-cancer properties

**Figure 8:**
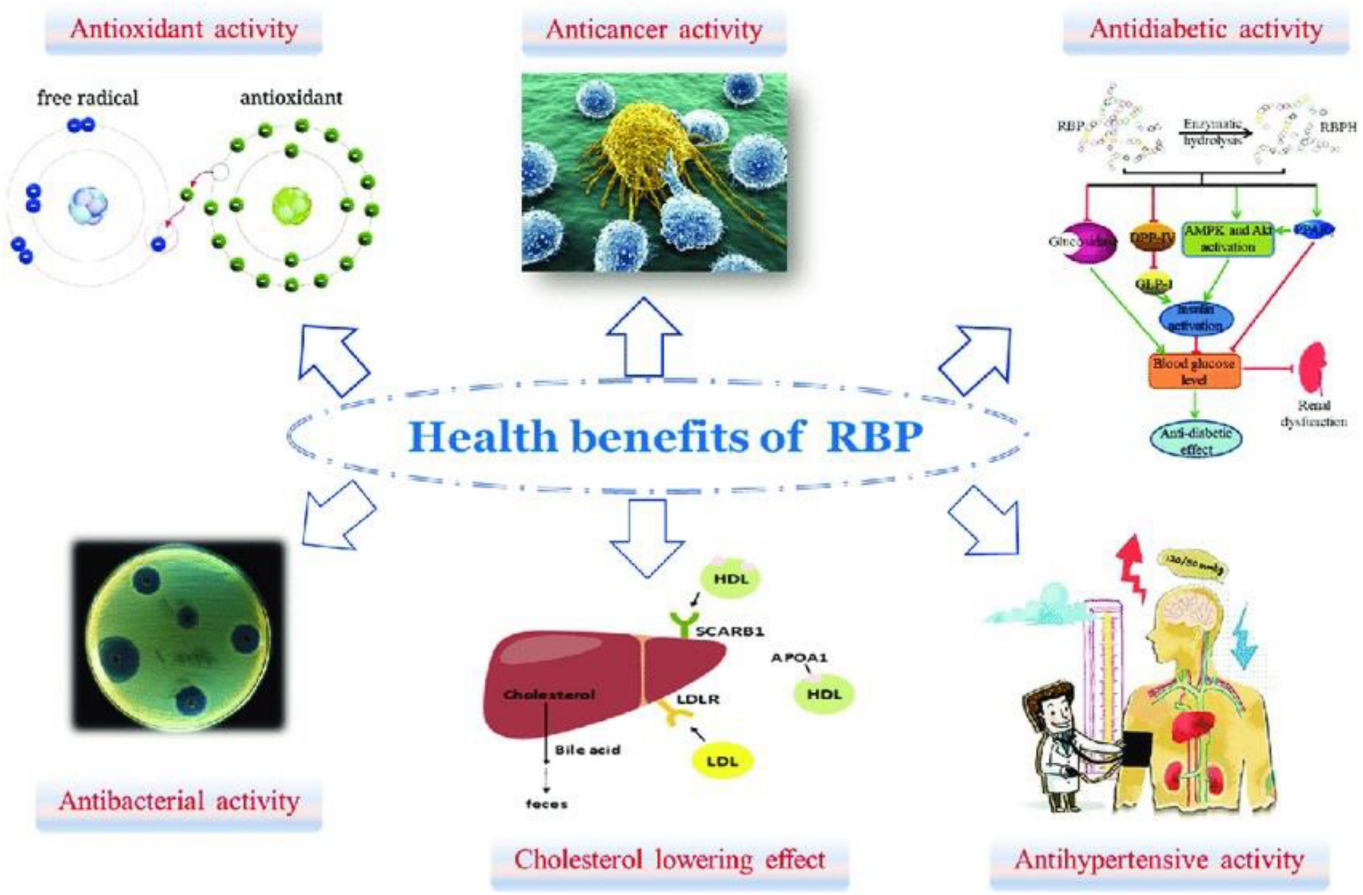
Health benefits of protein in bran rice.

Proteins, as essential functional components, exhibit significant anti-cancer properties through diverse mechanisms. Proteins, including bioactive peptides and enzymes, contribute to cancer prevention and therapy by modulating immune responses, inducing apoptosis, and inhibiting tumor cell proliferation and metastasis (**Figure 8**). The synergistic interactions may further amplify their anti-cancer effects, offering promising avenues for nutraceutical and therapeutic applications Phenolic compounds and lignans in rice and barley have been linked to reducing risks of certain cancers, including colon and breast cancer (Ahmed et al., 2025). Further research is needed to optimize their bioavailability, dosage, and clinical efficacy, paving the way for innovative cancer prevention and treatment strategies.

### 5.3 Immune support

Proteins play crucial roles as a functional component in supporting the human immune system. Proteins, particularly those containing essential amino acids, are vital for the production of antibodies, cytokines, and immune cells, ensuring proper immune response and defense against pathogens (Garcés-Rimón et al., 2022). The compounds contribute to a robust and balanced immune system, helping to prevent infections, reduce chronic inflammation, and improve overall health. Incorporating a diet rich in high-quality proteins and flavonoid-containing foods, such as lean meats, legumes, fruits, and vegetables, can be an effective strategy for maintaining optimal immune function and long-term well-being. Vitamins, minerals, and antioxidants in both cereals support immune function and overall health (Tripathy et al., 2025).

### 5.4 Hypoallergenic properties

Proteins, as a functional component, exhibit significant potential in enhancing hypoallergenic properties in the human body. Certain proteins, such as hydrolyzed or fermented peptides, can reduce allergenicity by breaking down allergenic epitopes, making them less recognizable to the immune system. Additionally, bioactive peptides may modulate immune responses, promote tolerance and reduce hypersensitivity reactions. The compounds can help mitigate allergic responses, offering a natural and dietary approach to managing allergies. Rice proteins are hypoallergenic and are often used in infant formulas and allergy-free diets (Tang et al., 2024).

### 5.5 Cholesterol-lowering effects

Dietary proteins, particularly those derived from plant sources (e.g., soy, legumes) and certain animal proteins (e.g., whey, fish), help reduce LDL (“bad”) cholesterol while promoting HDL (“good”) cholesterol through mechanisms such as enhanced bile acid excretion, modulation of lipid metabolism, and improved satiety leading to weight management (**Figure 8**). The synergistic action of these bioactive compounds supports cardiovascular health by improving lipid profiles, reducing oxidative stress, and mitigating atherosclerosis risk. Barley proteins have been shown to reduce cholesterol levels, contributing to cardiovascular health (Ho et al., 2016).

## 6 Technological properties

Gelation and emulsification in rice proteins exhibit excellent gelatin and emulsification properties, making them useful in food processing and product development (Sun et al., 2025). Viscoelasticity in barley proteins, particularly hordeins, contribute to the viscoelastic properties of dough, which is important for baking and brewing applications (Cappelli et al., 2020). Rice and barley are not only staple foods but also rich sources of functional components that offer numerous health benefits. Incorporating whole grain rice (e.g., brown rice, black rice) and barley into the diet can provide essential nutrients, antioxidants, and bioactive compounds that support cardiovascular health, glycemic control, gut health, and overall well-being. Further research is needed to explore the full potential of these functional components and their applications in functional foods. Rice and barley proteins are used in the development of functional foods, such as protein-enriched snacks, beverages, and supplements. As plant-based diets gain popularity, rice and barley proteins are increasingly used as alternatives to animal proteins. Genetic engineering offers a powerful approach to simultaneously enhance protein quality content in rice and barley by targeting metabolic pathways, regulatory genes, and protein-flavonoid interactions. There are some strategic approaches and specific genetic targets for both crops; dual-enhancement strategies, transcription factors (TF) engineering, and RNA interferences (RNAi) to block competing pathways. By strategically combining pathway engineering, transcription factor modulation, and protein optimization, rice and barley can be tailored to deliver high-quality protein simultaneously. This dual-biofortification approach could address “hidden hunger” (micronutrient + protein deficiencies) in staple crops. The future tools are using CRISPR-Cas9 for multiplex editing or synthetic biology to design fusion proteins (e.g., flavonoid-binding peptides).

## 7 Conclusions

The study of proteins in rice and barley reveals a complex picture that underscores their critical importance from both a biological and a human perspective. While both are staple cereals, their protein profiles exhibit distinct characteristics that define their unique value. In terms of composition, both cereals store nitrogen primarily in the form of prolamins; in rice, this includes both glutelins and prolamins, while in barley, the primary storage prolamins are the hordeins. However, a key distinction lies in their overall protein quality; rice proteins are renowned for their high lysine content and superior digestibility, making them a higher quality protein source. Barley, while generally higher in total protein content, is limited by its lower levels of essential amino acids like lysine. Underpinning these compositional differences is a highly coordinated biosynthetic process occurring within the developing endosperm. It involves the transcription and translation of specific gene families, followed by complex folding, modification, and deposition into protein bodies. The pathways for prolamin synthesis in the endoplasmic reticulum and glutelin synthesis involving the Golgi apparatus are particularly crucial, with rice’s unique dual-mode system for glutelin storage being a notable feature. This entire process is under precise gene regulation, orchestrated by a network of transcription factors (e.g., RISBZ and RPBF in rice) that respond to developmental cues and nitrogen availability. The spatial, temporal, and nutrient-dependent expression of prolamin and glutelin genes ensures the efficient sequestration of nitrogen into the grain, directly influencing the final protein composition and, consequently, the grain’s nutritional and functional properties. The functionality of these proteins extends far beyond seed germination. They are the primary determinants of the technological quality of grains. The type and ratio of storage proteins govern the physicochemical properties of flour, critically influencing end-use applications like the baking and malting quality of barley and the cooking and textural properties of rice. Finally, the nutritional significance of rice and barley proteins is profound. They are a vital source of dietary protein for billions of people. The high digestibility and balanced amino acid profile of rice protein make it exceptionally beneficial for human nutrition, often considered hypoallergenic. Barley protein, particularly when consumed as whole grain, contributes significantly to daily protein intake and is accompanied by the health benefits of beta-glucans. Ongoing research and biotechnological efforts, such as biofortification and genetic engineering, aim to further enhance the amino acid balance and content in both cereals to combat global malnutrition. Ultimately, the proteins in rice and barley represent a perfect nexus of agronomy, genetics, food science, and human health. Understanding their composition, biosynthesis, and regulation is not merely an academic pursuit but is essential for driving innovations in crop improvement, food processing, and nutritional security worldwide.
